# The Implausibility of Metabolic Cycles on the Prebiotic Earth

**DOI:** 10.1371/journal.pbio.0060018

**Published:** 2008-01-22

**Authors:** Leslie E Orgel

## Abstract

It has been suggested that complex reaction cycles, analogous to metabolic cycles, operated on the primitive Earth prior to the origin of genetic systems, but severe difficulties arise when these proposals are scrutinized from the standpoint of chemical plausibility.

Cycles occur widely in all branches of chemistry. The definition of a catalyst as an agent that facilitates the conversion of reactants to products without itself being changed almost guarantees that a catalyst can initiate successive “cycles” of the same reaction. Metabolic cycles are different. Strictly, they are by definition restricted to biochemistry. Like catalytic cycles, they too result in repeated conversions of substrates into products, but they involve much more complex sequences of chemical reactions. As far as I am aware, the formose reaction, which converts formaldehyde to a complicated mixture of products, including various sugars [1], is the only known nonenzymatic reaction sequence that is at all similar to a metabolic cycle, although the existence of one or two much simpler cycles has been established or made probable in the literature of prebiotic chemistry [2,3]. The possibility that reactions of hydrogen cyanide (HCN) might form the basis for a complex cyclic organization has been proposed [4], but there is as yet no experimental evidence to support this proposal.

If complex cycles analogous to metabolic cycles could have operated on the primitive Earth, before the appearance of enzymes or other informational polymers, many of the obstacles to the construction of a plausible scenario for the origin of life would disappear. If, for example, a complex system of nonenzymatic cycles could have made nucleotides available for RNA synthesis, many of the problems of prebiotic chemistry would become irrelevant. Perhaps a simpler polymer preceded RNA as the genetic material—for example, a polymer based on a glycerol-phosphate backbone [5] or a phosphoglyceric acid backbone. Could a nonenzymatic “metabolic cycle” have made such compounds available in sufficient purity to facilitate the appearance of a replicating informational polymer?

It must be recognized that assessment of the feasibility of any particular proposed prebiotic cycle must depend on arguments about chemical plausibility, rather than on a decision about logical possibility. Any reaction sequence that is allowed by thermodynamics could, in principle, be realized, given a sufficiently active and specific family of catalysts. Plants synthesize complex alkaloids, such as strychnine, from CO_2_, NH_3_, and reducing equivalents, so it must, in principle, be possible to achieve these syntheses starting from CO_2_, NH_3_, and H_2_, given a family of sufficiently active and specific prebiotic catalysts. However, few would believe that any assembly of minerals on the primitive Earth is likely to have promoted these syntheses in significant yield. Each proposed metabolic cycle, therefore, must be evaluated in terms of the efficiencies and specificities that would be required of its hypothetical catalysts in order for the cycle to persist. Then arguments based on experimental evidence or chemical plausibility can be used to assess the likelihood that a family of catalysts that is adequate for maintaining the cycle could have existed on the primitive Earth.

The metabolic cycles that have been identified by biochemists are of two kinds: simple cycles and autocatalytic cycles. The citric acid cycle, which brings about the oxidation of acetate to CO_2_ with the concomitant synthesis of ATP, and the urea cycle that results in the conversion of toxic NH_3_ to relatively harmless urea, are both examples of simple cycles. The initial step of the former cycle is the synthesis of citric acid from oxaloacetic acid and acetyl-CoA. After one turn of the cycle, acetate is completely “burned” to CO_2_ as one molecule of oxaloacetate is regenerated. The Calvin dark cycle and the reverse citric acid cycle, both of which result in the fixation of CO_2_ into important biochemical intermediates, are examples of autocatalytic cycles. The reverse (reductive) citric acid cycle (Figure 1) is initiated by the splitting of citric acid to give oxaloacetic acid and acetyl-CoA. After one turn of the cycle, two molecules of citric acid are formed, so long as no material is diverted from the cycle. That is why the cycle is described as autocatalytic—each molecule of citric acid introduced into the cycle results, after a turn of the cycle, in the generation of two molecules of citric acid. The proposal that the reverse citric acid cycle operated nonenzymatically on the primitive Earth has been a prominent feature of some scenarios for the origin of life [6–8].

**Figure 1:**
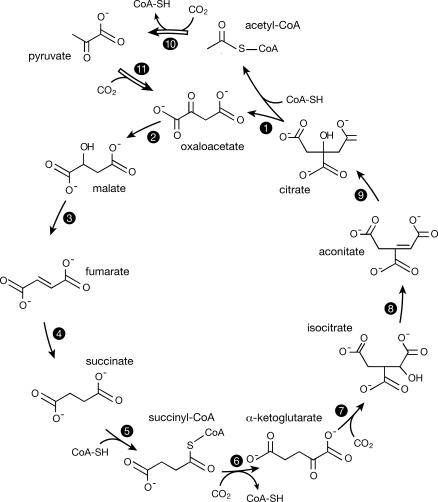
The Reverse (Reductive) Citric Acid Cycle Only the carbon species are shown, but the cycle also consumes four molecules of H_2_ and generates two molecules of H_2_O per turn. The main cycle (reactions 1–9) becomes autocatalytic if coupled to an epicycle that returns acetate to oxaloacetate (reactions 10 and 11).

A different kind of autocatalytic cycle, which has no analog in biochemistry, has been hypothesized by Stuart Kauffman to self-organize spontaneously whenever amino acids condense together to form peptides [9]. According to Kauffman, the catalytic properties of some of the members of a random-sequence mixture of peptides guarantee that a cyclic organization will emerge in which a small number of peptides will come to dominate the chemistry of the polymerization reaction. These peptides together with their subsequences will catalyze their own synthesis from monomeric amino acids and will constitute a cycle in which each peptide catalyzes the synthesis from smaller precursors of the next peptide of the cycle.

The main purpose of this Essay is to examine the plausibility of these and some related hypothetical nonenzymatic cycles. Could prebiotic molecules and catalysts plausibly have the attributes that must be assigned to them in order to make the self-organization of the cycles possible?

## The Reverse Citric Acid Cycle

### General considerations.

Figure 1 presents a diagram of the reverse citric acid cycle. Single-headed arrows are used in the diagram to indicate the direction in which the reaction pathways must be traversed, but it is important to realize that many of the reactions are reversible. Open arrows are used to differentiate the reactions that allow the cycle to become autocatalytic from those required for a simple catalytic cycle.

The reaction sequence in Figure 1, if one ignores the steps implied by the open arrows, results in the synthesis of acetic acid from CO_2_ and H_2_:

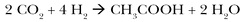



It is a catalytic cycle in which a complicated sequence of enzymatic reactions is used to bring about indirectly a reaction that looks simple on paper, but is not easily achieved in practice. Biotin, thiamine, and CoA are essential cofactors, and the completion of the reactions of the cycle is accompanied by the hydrolysis of at least two molecules of ATP. If there were no complications, each molecular exemplar of the cycle could produce sequentially an indefinitely large number of acetic acid molecules, given a sufficiently long time.

A catalytic reaction can be characterized by the rate of the reaction and the turnover number, that is, the average number of product molecules formed before the catalyst is deactivated. In a similar way, the cycle of reactions that we are discussing is characterized by a rate that is determined by the average cycling time and a total turnover number, which represents the average number of turns of the cycle that occur before the cycle is disrupted. The reduction of pyruvate to lactate, for example, is a side reaction that would be expected in a reducing environment [10]. If this reaction occurred irreversibly 50% of the time, each molecule of the cycle could catalyze, on average, the formation of only a single acetate molecule. If it occurred irreversibly only 1% of the time, then about 100 molecules of acetate would be formed.

Catalytic conversions must be completed in, at most, days to be useful in a laboratory setting, but the only external constraint on a useful prebiotic reaction is set by the time available on the primitive Earth for its completion. Unfamiliarly low reaction rates, therefore, may be acceptable for the reactions proposed in a prebiotic scenario [8]. The total turnover number, however, is always important. A reaction that catalyzes the production of one molecule of acetate per molecule of citrate degraded is certainly not useful, whereas a reaction that produces 100 might be, provided the cycle could occasionally be topped up by a small exogenous supply of one of its constituents. I have introduced this short treatment of the nonautocatalytic reverse citric acid cycle because it provides a useful introduction to the later discussion. It has not been suggested that the nonautocatalytic cycle alone was a source of prebiotic self-organization on the primitive Earth.

The inclusion of the reactions designated by the open arrows in Figure 1 completely changes the dynamics of the reverse citric acid cycle. If all reactions were completely efficient, each turn of the cycle would result in the formation of two oxaloacetate molecules starting from one, the second molecule being formed from four CO_2_ and five H_2_ molecules. To maintain the concentration of oxaloacetate and the other cycle components would then require an efficiency of only half the theoretical maximum efficiency. Fully half of the turns of the cycle could be diverted to side products, some or all of which might find use at a later stage of chemical evolution. The cycle could not survive if side reactions funneled off more than half of the cycle components irreversibly, because then the concentration of the cycle components would decline exponentially to zero.

The reverse citric acid cycle is self-sufficient in the sense that it can, in principle, persist in the absence of any substrates other than CO_2_ and H_2_. It would require external catalysts, presumably minerals, and almost certainly external cofactors and energy sources. However, the time available for self-organization could have been large, so if the cycle is stable, it might have operated slowly, for example, on spatially separated mineral catalysts [8]. However, the identification of a cycle of plausible prebiotic reactions is a necessary but not a sufficient step toward the formulation of a plausible self-organizing prebiotic cycle. The next, and more difficult step, is justifying the exclusion of side reactions that would disrupt the cycle.

The reverse citric acid cycle involves at least 11 distinct reactions. If each reaction proceeded independently, the completion of the cycle with 50% efficiency would require an average efficiency per reaction of over 90%. However, the reactions are not independent because each reaction is pulled toward completion by the use of its product as the input for the subsequent reaction of the cycle. In the absence of irreversible side reactions, even reactions that are intrinsically inefficient separately could combine together to make a working cycle. This can be illustrated by considering the conversion of isocitric acid to citric acid via aconitic acid (Figure 1, reactions 8 and 9). Any catalyst or family of catalysts that could convert isocitric acid to citric acid could also achieve the reverse reaction. Whatever the original input, one would finish with an equilibrium mixture, the composition of which is determined by thermodynamics. Suppose, for ease of exposition, that the equilibrium mixture contains 50% of each isomer. Then the removal of some citric acid by conversion to oxaloacetate and acetyl-CoA would be followed by a compensatory conversion of isocitric acid to citric acid. This would ultimately allow all of the isocitric acid to be used up. This, of course, would be true only in the absence of disruptive side-reactions of citric acid, isocitric acid, or aconitic acid.

### The reactions of the reverse citric acid cycle.

The reverse citric acid cycle involves a number of fundamentally different kinds of chemical transformations:

Reversible dehydration reactions that convert malic acid to fumaric acid (reaction 3) and equilibrate isocitric acid with citric acid via aconitic acid (reactions 8 and 9). At equilibrium, malate is in about 3-fold excess over fumarate at moderate temperatures in aqueous solution [11]. The uncatalyzed equilibration is very slow: the reaction is usually studied in concentrated acid (or alkali) at temperatures well above 100 °C. The half-time to reach equilibrium at room temperature and moderate pH in the absence of a catalyst must exceed 10,000 y [12,13]. The uncatalyzed equilibration of isocitrate with citrate via aconitate is likely to proceed on roughly the same timescale.
The conversion of succinic acid to succinyl-CoA (reaction 5) at the expense of ATP, and probably a similar conversion of citric acid to a CoA derivative (see below). Presumably the prebiotic reaction would involve an inorganic polyphosphate and a simple sulfhydryl compound.
A lyase reaction in which citric acid is split to oxaloacetic acid and activated acetate (reaction 1). In the enzymatic reaction, ATP is used as a source of energy, and the acetate is made available as a derivative of CoA. It seems likely that the more primitive pathway for this reaction involved two distinct enzymes, one that attached citric acid to CoA and a second that split the adduct to oxaloacetic acid and activated acetate [14].
A reaction in which the ketoacid—oxaloacetic acid—is reduced to the corresponding hydroxyacid—malic acid (reaction 2). This is a relatively easy reduction reaction, and it is reasonable to assume that it could proceed quickly in the presence of a mineral catalyst. A similar reduction occurs in the second step of reaction 7.
The reduction of the double bond of fumaric acid to give succinic acid (reaction 4). This is another reaction that might be expected to proceed rapidly in the presence of a suitable mineral catalyst, possibly the same catalyst as the one that reduces oxaloacetic acid.
The carboxylation of the ketoacids pyruvic acid and a-ketoglutaric acid to give oxaloacetic acid (reaction 11) and, following reduction, isocitric acid (first step of reaction 7), respectively. The reversal of these carboxylation reactions are well known in organic chemistry and proceed rapidly without the need for catalysts. The law of detailed reversibility implies that the forward carboxylation reactions must also reach equilibrium rapidly. However, the equilibria are so strongly in favor of the decarboxylated products that it seems essential that the reaction should be driven forward by coupling to some other energy-yielding reaction. In biochemistry, the carboxylation of pyruvic acid is coupled, through biotin, to the hydrolysis of pyrophosphate bonds of ATP. To realize the prebiotic counterpart, some equivalent coupling to an exergonic reaction would be necessary; for example, coupling to the hydrolysis of an inorganic pyrophosphate or polyphosphate. This would presumably require additional specific catalysts, because otherwise, phosphate groups would be introduced globally and would destroy the cycle. A cofactor equivalent to biotin, possibly urea that could be regarded as an acyclic analog of biotin, would almost certainly be needed.
The reductive carboxylations of acetic acid and succinic acid, via CoA derivatives, to give pyruvic acid and a-ketoglutaric acid, respectively (reactions 10 and 6). These are chemically very difficult reactions [15]. It might be advantageous to abandon CO_2_ as the unique carbon input to the prebiotic cycle and accept CO as a secondary input for these steps. There is evidence that pyruvic acid can indeed be formed in relevant ways from CO in the presence of metal sulfide catalysts at high temperatures and pressures [16].

At the very least, six different catalytic activities would have been needed to complete the reverse citric acid cycle. It could be argued, but with questionable plausibility, that different sites on the primitive Earth offered an enormous combinatorial library of mineral assemblies, and that among them a collection of the six or more required catalysts could have coexisted.

### Side reactions that might disrupt the reverse citric acid cycle.

While enzymes discriminate readily between very similar substrates, such discrimination is rare, but not impossible, in reactions catalyzed by small molecules or mineral surfaces. There are a few places where a catalyst would need to be specific with respect to the components of the reverse citric acid cycle in the sense that it facilitated the transformation of one component of the cycle while failing to transform another component of the same chemical class.

In the first half of the cycle, fumaric acid is reduced to succinic acid, while in the second half the corresponding reduction must not occur, so that aconitic acid can hydrate to citric acid. This does not necessarily present a serious problem, but citric acid would need to be split to oxaloacetic acid and activated acetic acid fast enough to avoid this disruptive reaction. Another potential complication, the splitting of isocitric acid via a CoA derivative to succinic acid and glyoxylic acid, would return succinic acid to the cycle. This would lower the efficiency of the cycle because a proportion of the acetate or oxaloacetate entering the cycle would be shunted to glyoxylic acid. Similarly, the splitting of malic acid to glyoxylic acid and acetic acid would return acetic acid to the cycle, but only at a cost in efficiency.

A possibly more serious problem is posed by the difficult reductive carboxylation reactions. If they proceed with acetic acid and succinic acid as inputs they might be expected to accept malic acid as an equivalent input. This reaction would move material irreversibly out of the cycle, so one must postulate a specific catalyst that discriminates between succinic and malic acid.

Some of the side reactions leading to molecules outside the cycle have already been discussed at length [10], so they will not be treated in detail here. Among the most troublesome are the reductions of pyruvic acid to lactic acid and of a-ketoglutaric acid to the corresponding hydroxyacid. Under almost any circumstance, a non-specific catalyst able to reduce oxaloacetic acid, oxalosuccinic acid, and fumaric acid would completely disrupt the cycle by also reducing pyruvic acid and a-ketoglutaric acid [10]. One needs, therefore, to postulate highly specific catalysts for these reactions. It is likely that such catalysts could be constructed by a skilled synthetic chemist, but questionable that they could be found among naturally occurring minerals or prebiotic organic molecules. The carboxylation reactions would also require specific catalysts to avoid the diversion of acetate to malonate, the reaction that initiates, among other pathways, the hydroxypropionate cycle for CO_2_ fixation.

The citrate lyase reaction presents severe difficulties in this context. The conversion of a carboxylic acid group to a thioester is a general reaction that, if it occurs with succinic acid and citric acid, would be expected to occur with almost any of the molecules involved in the reverse citric acid cycle. In many cases this would lead to potentially disruptive side reactions, and would always lead to non-productive use of the prebiotic activating agent. Two highly specific catalysts would clearly be needed to overcome this difficulty.

The above discussion has mainly considered potential side reactions that occur as enzyme-catalyzed reactions in biochemistry. There are many other reactions familiar to organic chemists that would also be troublesome. It is clear that the existence of a sequence of catalyzed reactions that would constitute an autocatalytic cycle is a necessary condition for the cycle to function in a sustained way, but it is not a sufficient condition. It is also necessary that side reactions that would disrupt the cycle be avoided. It is not completely impossible that sufficiently specific mineral catalysts exist for each of the reactions of the reverse citric acid cycle, but the chance of a full set of such catalysts occurring at a single locality on the primitive Earth in the absence of catalysts for disruptive side reactions seems remote in the extreme. Lack of specificity rather than inadequate efficiency may be the predominant barrier to the existence of complex autocatalytic cycles of almost any kind.

Smith and Morowitz contemplate a series of spatially separated mineral catalysts as the facilitators of the reverse citric acid cycle [8]. Wächtershäuser suggested that a related sequence of reactions occurs as a surface metabolism on some naturally occurring metal sulfide mineral [6]. While the details of the two proposals are different, the difficulty of achieving all of the required reactions while avoiding all of the likely side reactions seems at least as formidable in Wächtershäuser's scenario as in that of Smith and Morowitz. In a very recent paper [17], Wächtershäuser no longer attributes importance to surface metabolism and the reverse citric acid cycle, replacing them as a source of self-organization by “metabolic reproduction, evolution, and inheritance by ligand feedback”. Unfortunately he never explains, even in outline, how this mechanism could lead to the synthesis of the aminoacyl-nucleotide conjugates that seem to be an essential feature of the proposal.

### A digression on enzymatic carbon dioxide fixation.

In addition to the reverse citric acid cycle, there are three other well-recognized biochemical pathways for CO_2_ fixation: the Calvin cycle, the hydroxypropionate cycle, and the reductive acetyl-CoA linear pathway. Photosynthesis involving the Calvin cycle is responsible for most carbon fixation on Earth today, whereas a nonphotochemical Calvin cycle is widely distributed among prokaryotes. It is interesting to compare the kind of chemistry involved in the Calvin cycle with that involved in the reverse citric acid cycle.

In both cycles, almost all of the molecules involved carry two or more negative charges. In the Calvin cycle, the great majority of these charges are provided by phosphate groups, but in the reverse citric acid cycle, carboxylate groups are the only sources of negative charge. Furthermore, the only reduction that occurs in the Calvin cycle—the conversion of 3-phosphoglyceric acid to glyceraldehyde-3-phosphate—occurs via an acylphosphate intermediate. Reduction in the reverse citric acid cycle never involves a preliminary phosphorylation. Enzymes that use transition metal ions or iron-sulfur clusters play an important role in the reverse citric acid cycle, but are absent from the Calvin cycle, which uses Mg^2+^ and occasionally Zn^2+^ cofactors in its enzymes. It seems plausible, therefore, that the enzymes of the reverse citric acid pathway evolved in a region rich in transition metal ions and sulfur, whereas those of the Calvin cycle evolved where phosphate and magnesium were abundant. Presumably, one of these two cycles arose before the other. Is it possible to determine which came first by using information on biosynthetic pathways and genomics data? A decision on this question, though not directly relevant to the origin of life, would be of the greatest importance for understanding the history of protein-based metabolism on the early Earth.

Any suggestion that the Calvin cycle self-organized without the help of enzymes faces difficulties analogous to those already discussed in connection with the reverse citric acid cycle. There are only two intrinsically difficult reactions: the reduction of 3-phosphoglyceric acid to glyceraldehyde-3-phosphate and the uphill carboxylation of ribulose-1,5-diphosphate, which is enabled by the highly exothermic fission of the adduct into two molecules of 3-phosphoglyceric acid. However, the subsequent steps that convert a glyceraldehyde-3-phosphate derivative back to ribulose-1,5-diphosphate, although not particularly demanding individually, could only be achieved with a series of highly specific catalysts.

## Small-Molecule Cycles

The only autocatalytic cycle that has been demonstrated experimentally is that involved in the formose reaction—the polymerization of formaldehyde to give a notoriously complex mixture of products, including ribose, the organic component of the backbone of RNA. This reaction was discovered in the 19th century and has been investigated in some depth [1]. The presumed core cycle [18] results from each glycolaldehyde molecule that enters the cycle leading to the synthesis of up to two glycolaldehyde molecules at the end of the cycle. This exponential growth of the concentration of molecules that include carbon–carbon bonds has particularly dramatic consequences because the dimerization of formaldehyde to glycolaldehyde does not occur directly. The initiation of the cycle, therefore, depends on the presence of very small amounts of glycolaldehyde or related impurities in the formaldehyde solution. Under many circumstances, the formation of significant amounts of complex products occurs only after many hours, the time needed to complete many rounds of amplification of the impurities by means of the autocatalytic cycle. In such cases there is a long induction period during which nothing visible happens, followed by a short reaction period during which colored products are formed. Despite some successes [19–21], it is still not possible to channel the formose reaction in such a way as to produce ribose in substantial yield.

I am indebted to Albert Eschenmoser for drawing my attention to a paper by Alan Schwartz and coworkers in which they demonstrate a simple (not autocatalytic) cyclic production of HCN tetramer from HCN in the presence of formaldehyde [2]. The initial rate of production of tetramer from HCN monomer is increased almost 100-fold when 0.1 M formaldehyde is introduced into a 0.2 M solution of HCN. A reaction of this kind presumably explains the increase in adenine yield that is obtained from eutectic solutions of HCN when formaldehyde is added to the solution [22]. This is the only reaction cycle of which I am aware that could have a marked effect on the efficiency of a potentially important prebiotic synthesis. Even in this case the synthesis of tetramer is accompanied by the synthesis of a complex mixture of other products.

Arthur Weber implies an autocatalytic cycle of as yet unknown mechanism in a 2007 publication [3]. Treatment of glyceraldehyde with NH_3_ in aqueous solution results in rearrangement to pyruvaldehyde and also leads to the formation of a large number of nitrogen-containing products. If the product mixture from such a reaction is added back to a fresh aliquot of glyceraldehyde solution, the rate of formation of pyruvaldehyde is greatly enhanced. Clearly, one or more of the products of this reaction are an effective catalyst for the reaction, the signature of an autocatalytic cycle. It seems at least possible that relatively simple cycles of this kind might be important sources of prebiotic material.

## Cycles and the Evolution of Complexity

So far, I have treated autocatalytic chemical cycles as particularly sophisticated prebiotic reaction sequences that result in the amplified production of potentially useful organic molecules. However, it has sometimes been implied or claimed that such cycles are not only stable, but also are capable of evolving to form nonenzymatic networks of great complexity. Genetic materials are then seen as late additions to already fairly complex evolved life forms [23]. According to this view, a genetic material merely adds stability to systems that already have a substantial “information content.” It is not easy to translate these intuitions into schemes that can be examined critically, but I will try.

A cycle—for example the reverse citric acid cycle—does not seem capable of evolving in any interesting way without becoming more complex. It is of course true that if the conditions under which the cycle operates are changed, for example by changing the temperature or the available catalysts, the kinetics of the cycle will respond. This, however, is true for any reaction or reaction sequence, and one could not usefully claim that the dependence of the rate of a reaction such as ester hydrolysis on reaction conditions is a form of evolution.

The evolution of any substantial additional complexity of a cycle, therefore, must depend on the appending of further reaction sequences to those present in the core cycle. One way of achieving something useful might be to use one of the constituents of the core cycle as the starting point of a second independent autocatalytic cycle. If malic acid, for example, could initiate a catalytic cycle of CO_2_ fixation and reduction, then the reverse citric acid cycle could have an analog of a relay amplification station and might, in addition, supply further compounds that are useful in prebiotic chemistry. Given the difficulty of finding an ensemble of catalysts that are sufficiently specific to enable the original cycle, it is hard to see how one could hope to find an ensemble capable of enabling two or more. Another suggestion that might be explored is the possibility of a side reaction generating a catalyst for one of the reactions of the core cycle, as suggested by the work of Weber discussed above [3]. This might reduce the dependence of the core cycle on external catalysts. However, neither of these possibilities, nor any others with which I am familiar explains how a complex interconnected family of cycles capable of evolution could arise or why it should be stable.

If a complex system of evolvable nonenzymatic cycles that did not depend on residue-by-residue replication could be shown to be feasible, it would mark a breakthrough in origin-of-life studies. What is essential, therefore, is a reasonably detailed description, hopefully supported by experimental evidence, of how an evolvable family of cycles might operate. The scheme should not make unreasonable demands on the efficiency and specificity of the various external and internally generated catalysts that are supposed to be involved. Without such a description, acceptance of the possibility of complex nonenzymatic cyclic organizations that are capable of evolution can only be based on faith, a notoriously dangerous route to scientific progress.

## Peptide Cycles

In a paper titled “Autocatalytic Sets of Proteins” [9], Kauffman has proposed a theory of peptide self-organization that specifically excludes residue-by-residue recognition. The theory is mathematical in nature, but I will try to explain the central idea without going into mathematical detail. Kauffman envisages a system in which the input consists of an aqueous solution of a mixture of amino acids. He assumes that amino acids are capable of reacting together spontaneously to form peptides and that peptides can undergo spontaneous hydrolysis at any internal amide bond. Kauffman also assumes that there is a small probability, *P*, that a randomly chosen peptide will catalyze the ligation of any two other randomly chosen peptides. He next considers how likely it is that a randomly chosen set of peptides of length *M* can be formed from monomers and arranged to yield a closed cycle, where closure means that each peptide catalyzes the formation of the next peptide in the cycle from one or more pairs of smaller peptides. Closure, importantly, also requires that the peptides of the cycle and their subsequences collectively catalyze the specific synthesis of the components of the cycle from amino acids.

It is obvious that if *P* is small, the probability of closing the cycle with a randomly chosen set of peptides must be very small, and gets smaller as the number of peptides in the cycle increases. Nonetheless, Kauffman shows that as *M* gets larger, the number of possible choices of random sets of peptides increases faster than the probability decreases that any one such set will form a closed cycle. Consequently, as *M* increases, it must eventually reach a value for which the existence of a closed cycle is virtually certain. This proof establishes that, given any value of *P*, it is always possible to write down on paper a cycle of peptides that is closed in the sense envisaged by Kauffman. The minimum value of *M* required to make the existence of a closed cycle probable, of course, depends on the assumed value of *P*, and would also depend on the number of different amino acids in the mixture. Kauffman assumes throughout his illustrative examples that there are just two amino acids, A and B, but the theory could be generalized to mixtures of an arbitrary number of amino acids.

Kaufmann takes it for granted that if it is possible to write down on paper a closed peptide cycle and a set of catalyzed ligations leading from monomeric amino acids to the peptides of the cycle, then that cycle would self-organize spontaneously and come to dominate the chemistry of a reaction system. This, as I will discuss below, is unlikely because peptide molecules do not have the properties that Kauffman assigns to them. I will illustrate the difficulties in the context of the system of two amino acids, A and B, but I have also explored a number of alternative systems with different numbers of amino acids or with inputs of random families of short peptides, and I find that they all encounter similar or more severe difficulties. Before beginning the discussion of the core of the theoretical argument, it is necessary to clear up a few misunderstandings concerning the thermodynamics of peptide bond formation.

Kauffman assumes that, in sufficiently concentrated solution, the naturally occurring amino acids or some subset of them would condense spontaneously to form a mixture of long peptides in substantial yield. In practice, this would not happen. The mistaken conclusion arises in part from assuming a value of 1.2 kcal/mol for the free energy of hydrolysis of a peptide bond, rather than a value of about 2.4 kcal/mol, as suggested by recent experiments [24]. A more serious misunderstanding concerns the effect on peptide bond formation of replacing a 1 M solution of a single amino acid by a solution of a mixture of amino acids. Kaufmann's conclusions would only be true if each amino acid in the mixture was present at a concentration of 1 M. If the total concentrations of the different amino acids sums to 1 M, as is assumed in his model, the replacement of a single amino acid by a mixture of amino acids would not result in a substantial increase in the amounts of long peptides formed.

These misunderstandings are not fatal for the theory, but they do require a reformulation of the proposed experiment. The reaction mixture must contain, in addition to amino acids, a coupling agent that provides the free energy needed to drive peptide bond formation. This does not affect the mathematical argument, but adds to the difficulty of proposing a relevant prebiotic system to which the mathematical treatment might apply. However, similar difficulty arises for all theories of the origin of life that involve the formation of polypeptides or polynucleotides. It could only be avoided by proposing a series of monomers, such as aminoaldehydes, that polymerize spontaneously, but the difficulty of finding a prebiotic synthesis of suitable monomers then becomes severe.

One can restate Kauffman's claim simply as follows: if a solution containing two amino acids—for example a mixture of l-arginine and l-glutamic acid or of l-alanine and l-histidine—is allowed to reach a steady state in which spontaneous or peptide-catalyzed hydrolysis of pre-existing peptide bonds is balanced by the uphill formation of new peptide bonds, a cycle involving peptides of substantial length will come to dominate the chemistry of the solution. The calculated length of the peptide constituents of the cycle, of course, depends on the assumed value of *P*, the probability that a randomly chosen triplet of peptides is a “catalytic triplet,” and on a number of other details of the model. Kauffman suggests that a value of *P* equal to or greater than 10^−9^ is likely and that a value of *M* no greater than 13 would then follow. Here I will discuss the likelihood that a cyclic steady state of this kind could be stable, but I will not attempt the much more difficult task of discussing the way in which a cycle of this kind might become established. I will assume that *P* = 10^−7^ and that *M* is consequently 10, as suggested in Table 2 of Kauffman's paper [9].

Ghadiri and his coworkers have demonstrated experimentally that peptide cycles of the type envisaged in Kauffman's theory are possible. They first showed that peptides of length 32 that have been carefully designed to self-associate to form stable coiled-coils will facilitate the ligation of their N-terminal and C-terminal subsequences. This shows that the self-replication of peptides is possible [25]. In later work they demonstrated the self-organization of networks of ligation reactions when more than two carefully designed input peptides are used [26]. These findings, however, cannot support Kauffman's theory unless the prebiotic synthesis of the specific 15mer and 17mer input peptides from monomeric amino acids can be explained. Otherwise, Ghadiri's experiments illustrate “intelligent design” of input peptides, not spontaneous self-organization of polymerizing amino acids. This takes us to the core of the problem: do sets of peptides of length approximately ten exist that have the ability to constitute a cycle and, even more importantly, could they and their subsequences specifically catalyze their own formation from a mixture of amino acids?

Both explicit and implicit assumptions of the peptide cycle theory present difficulties. The most obvious concerns the assumed value of *P*, coupled with the assumed specificity of the ligation reactions. In the illustrative example, *P* is explicitly assumed to have a value in the range of 10^−6^–10^−9^ for catalyzed ligations, and it is implicitly assumed that catalysis is negligible for ligation reactions that are not involved in the cycle. Sufficiently efficient catalysis could possibly be demonstrated for peptides rich in histidine, for example, because histidine is an imidazole derivative and imidazole derivatives are often excellent general acid–base catalysts. In fact, any peptide containing enough histidine residues might catalyze indiscriminate peptide ligation, so *P* could approach unity. In that case, Kauffman's criteria would be met because any set of two or more peptides of length *M* containing histidine could be synthesized from amino acids and written as a closed cycle. However catalysis of this kind would increase the rate at which all peptides accumulate, but would not lead to self-organization. Instead, it would support a faster synthesis of a complex, nonorganized mixture of peptides. Clearly, self-organization requires catalysis that is not only sufficiently efficient but also sufficiently sequence-specific.

The catalytic properties of enzymes are remarkable. They not only accelerate reaction rates by many orders of magnitude, but they also discriminate between potential substrates that differ very slightly in structure. Would one expect similar discrimination in the catalytic potential of peptides of length ten or less? The answer is clearly “no,” and it is this conclusion that ultimately undermines the peptide cycle theory. One must therefore consider the discrimination that would be needed to make the cycle theory plausible and then explain why short peptides are extremely unlikely to exhibit the necessary catalytic specificities.

I take as an example a peptide cycle that is composed of five decamers because this seems to be the type of cycle that Kauffman envisages. Then the complete self-organized system for the operation of the cycle and the synthesis of its constituents from monomeric amino acids can only include the five decamer sequences, ten nonamer subsequences, 15 octamer sub-sequences, and so on for all subsequences down to the dimers. The task that this set of potential catalysts must achieve, in addition to the operation of the cycle, is the synthesis of the 45 specific peptide bonds needed to create a set of five decamers of the required sequences. The side reactions that must be avoided or at least minimized are all ligation reactions that lead to products outside the cycle. What kind of peptides could have such remarkable catalytic specificity?

The specificity of protein catalysis is dependent on the stable three-dimensional structures of enzymes and enzyme-substrate complexes. Highly specific catalytic activity could only be expected from short peptides if they, too, adopted stable structures either alone or in association with potential substrates. In fact, short peptides rarely form stable structures, and when they do, the structures are only marginally stable. The synthesis of a decapeptide that would catalyze the ligation in the correct order of two particular pentapeptides out of a mixture of ten pentapeptides that are required to form the five cycle components, while failing to bring about any of the other possible ligations, would present an extremely difficult challenge to peptide chemistry. It seems certain that the additional requirement that this peptide should also catalyze specifically many of the reactions leading from amino acids to the pentamer precursors of the decamers of the cycle could never be met. Of course, the decamers need not be formed only from pairs of pentamers, but the difficulties are no less severe for more complex synthetic networks.

There are a number of possible ways in which this difficulty might be circumvented, but none seems relevant to the origin of life. Perhaps the members of “catalytic triplets” are not randomly distributed among peptide triplets but are strongly dependent on some generic feature of the sequences. Suppose, for example, that all peptides with a repeating (AB)*_n_* (where *n* = 1, 2, 3, …) sequence catalyze the addition of an A reside to a peptide terminating in B or the addition of a B residue to a peptide terminating in A, but never catalyze the addition of A to A, or B to B. Then alternating AB peptides might indeed come to dominate the reaction products formed from A and B. This does not seem very useful in the context of molecular evolution, nor is it clear that any peptides formed from potentially prebiotic amino acids would show the required specificity. More complex proposals along these lines need to be supported by experimental evidence or compelling theoretical arguments based on the known properties of amino acids and peptides. Claims that they exist cannot be taken seriously without support from experimental or theoretical chemistry.

A different and more interesting class of solutions to the problem might depend on finding very short peptides that adopt stable conformations either through self-association or through association with a structured surface. Recent work on the x-ray structures of amyloids provides a relevant background [27]. Peptides as short as tetramers, for example the peptide Asn-Asn-Gln-Gln, form long fibrils. These stable structures are formed by the very close packing of pairs of identical ß sheets. The sheets associate together along the fiber axis by hydrogen bonding. There is already evidence that amyloid fibrils formed by longer peptides will catalyze the ligation of subsequence peptides to form more of the full-length peptide, a form of self-replication [28]. It is not impossible that some amyloid fiber based on a very short peptide, say a tetramer, would catalyze its own extension from a mixture of activated amino acids. Even if such systems exist, their relevance to the origin of life is unclear. It is unlikely, therefore, that Kauffman's theory describes any system relevant to the origin of life.

## Conclusions

The demonstration of the existence of a complex, nonenzymatic metabolic cycle, such as the reverse citric acid, would be a major step in research on the origin of life, while demonstration of an evolving family of such cycles would transform the subject. In view of the importance of the topic, it is essential to subject metabolist proposals to the same kind of detailed examination and criticism that has rightly been applied to genetic theories [29,30]. In the case of these latter theories, an appraisal of their plausibility can be based on a substantial body of experimental work. In the case of the former, because little experimental work has been attempted, appraisal must be based on chemical plausibility.

Almost all proposals of hypothetical metabolic cycles have recognized that each of the steps involved must occur rapidly enough for the cycle to be useful in the time available for its operation. It is always assumed that this condition is met, but in no case have persuasive supporting arguments been presented. Why should one believe that an ensemble of minerals that are capable of catalyzing each of the many steps of the reverse citric acid cycle was present anywhere on the primitive Earth [8], or that the cycle mysteriously organized itself topographically on a metal sulfide surface [6]? The lack of a supporting background in chemistry is even more evident in proposals that metabolic cycles can evolve to “life-like” complexity. The most serious challenge to proponents of metabolic cycle theories—the problems presented by the lack of specificity of most nonenzymatic catalysts—has, in general, not been appreciated. If it has, it has been ignored. Theories of the origin of life based on metabolic cycles cannot be justified by the inadequacy of competing theories: they must stand on their own.

The situation with respect to chemical cycles unrelated to those involved in contemporary metabolism is different. At least one well-established autocatalytic cycle, the core of the formose reaction, is understood reasonably well [1,18] and, as discussed previously, there is experimental support for the existence of one or two other simple cycles [2,3]. This suggests that there may be more cycles to be discovered, and they could be relevant to the origin of life. The recognition of sequences of plausible reactions that could close a cycle is an essential first step toward the discovery of new cycles, but experimental proof that such cycles are stable against the challenge of side reactions is even more important.

Proposals involving complex metabolisms that are stable even in the absence of informational polymers usually are linked to the context of hydrothermal synthesis in the deep sea vents or some equivalent environment. Such linkage, however, need not be an essential feature of these theories [31]. A metabolist theory based on the self-organization of the Calvin cycle, for example, would be a logical possibility, although not necessarily an attractive one. Conversely, a theory in which metal sulfide–catalyzed reactions provided some or all of the organic molecules needed for the formation of a primitive genetic system would have many attractive features. A number of prebiotic syntheses catalyzed by transition metal sulfides under hydrothermal conditions have already been reported [16,17], and this is now an active area of research. It is important to realize that recognition of the possible importance of prebiotic syntheses that could occur hydrothermally does not necessitate a belief in their ability to self-organize.

The prebiotic syntheses that have been investigated experimentally almost always lead to the formation of complex mixtures. Proposed polymer replication schemes are unlikely to succeed except with reasonably pure input monomers. No solution of the origin-of-life problem will be possible until the gap between the two kinds of chemistry is closed. Simplification of product mixtures through the self-organization of organic reaction sequences, whether cyclic or not, would help enormously, as would the discovery of very simple replicating polymers. However, solutions offered by supporters of geneticist or metabolist scenarios that are dependent on “if pigs could fly” hypothetical chemistry are unlikely to help. _
